# Influence of 8-Week Aerobic Training on the Skin Microcirculation in Patients with Ischaemic Heart Disease

**DOI:** 10.1155/2020/4602067

**Published:** 2020-01-07

**Authors:** Renata Szyguła, Monika Wierzbicka, Grażyna Sondel

**Affiliations:** The Witelon State University of Applied Sciences in Legnica, Faculty of Health Science and Physical Education, Legnica, Poland

## Abstract

**Materials and Methods:**

In the study, 48 men took part with a stabilized and pharmacologically controlled ischaemic disease. The participants were randomly divided into two groups with 24 people in each of them. The research group participated in an aerobic march training. The march was taking place 3 times a week for 30–40 minutes over a period of 8 weeks. In the time of training, the subjects did not practise any other physical activity for 8 weeks. The measurement of skin microcirculation was done by using the laser Doppler flowmeter estimating the values of regular flow and the reactions provoked in response to occlusion and temperature. Signal frequency was also analysed which was received by means of laser Doppler flowmetry in the range from 0.01 to 2 Hz during the regular flow.

**Results:**

During the first measurement in relation to the initial values, a decrease in body mass was noted by 2.21 kg on average as well as reduction of systolic and diastolic pressure by 10.4 mmHg and 3.68 mmHg, respectively. The regular flow (RF) increased after the training by 2.21%. The provoked reactions were as follows: hyperemic (PRHmax): an increase occurred by 8.76% and hyperthermic (THmax): an increase occurred by 5.38%. The time needed to achieve PRHmax was reduced by 42% and to achieve THmax, by 22%. The heart rhythm and the signal strength of neurogenic rhythm decreased by approximately 8% and 24%, respectively. The signal strength of endothelial rhythm increased by 19%. In the second measurement, a recourse was noted in the values of indicators under investigation, which were assuming values close to the initial ones. In the control group, the measurement values did not change significantly.

**Conclusions:**

8 weeks of systematic aerobic training provides a significant improvement of endothelium functioning, expressed by reactivity improvement in skin microcirculation in patients suffering from ischaemic heart disease. It points to aerobic training as a nonpharmacological effective cardioprotective factor. The improvement effects of skin vascular bed functioning in the group of patients with IHD are impermanent, and they disappear after the period in which patients did not exercise physical activity.

## 1. Introduction

Ischaemic heart disease (IHD), or in other words, coronary artery disease (CAD), involves several clinical symptoms resulting from the surplus of demand over supply of aerobic and nutrient substrates in the myocardial cells. The consequence of IHD is acute coronary incidents, often leading to death. IHD and its complications, despite progress in the invasive and noninvasive treatment of this disease, are a huge problem of western societies [1, 2]. It is also alarming that increasingly more often young people at the age of 20–30 years suffer from this disease [3].

It was proved that coronary atherosclerosis, which in 90% of cases, is the cause of IHD, correlates significantly with dysfunctioning of vascular endothelium [4, 5]. It was also observed in patients with clinical symptoms of IHD with patent coronary arteries. It was confirmed that disorders of vascular endothelium in microcirculation are responsible for this state and not the advanced atherosclerotic process in large arteries [4, 6–8].

Investigating organ microcirculation, including the coronary one, is difficult and risky. However, Holowatz et al. [9] proposed that generalized systematic microcirculation functions are represented by easier accessible cutaneous placenta. Therefore, many studies of skin microcirculation reactivity were used for diagnosing and interpreting systemic disorders of microcirculation and mechanisms of their formation [10–12].

Impairment of vascular functions in the skin is considered to be an early predictor of developing cardiovascular diseases and an accepted IHD risk factor [10, 13, 14].

IHD risk factors are generally known, and it seems that the most significant one is the widely understood life style, especially the degree and the quality of physical activity. It was demonstrated that endothelium dysfunction in people with IHD is correlated with a lower level of physical activity [15]. It was proved that systematic physical effort lowers mortality and frequency of hospitalization and improves the quality of life in patients with ischaemic heart disease [16]. Possibly, the positive influence mechanism of physical activity on blood vessels results from an increase in laminar blood flow during effort. The increased flow generates the shear force, the so-called “shear stress,” which results in releasing vasoconstrictor factors, mainly nitric oxide (NO,) both while training, as well as after it [17, 18]. It is also believed that physical training may cause a reduction in the level of vasoconstrictive factors such as angiotensin II (Ang II) and endothelin (ET-1) [19–21]. The degree and quality of changes occurring in the vessels depend on the amount and intensity of the training [22]. Tinken et al. [18] demonstrated that in people, shear forces are different in the case of aerobic exercise and anaerobic exercise. Reverse flow occurring during anaerobic exercise is a negative stimulus for the vascular endothelium [23, 24]. Even short-term exercises of great intensity increase the reverse shear forces, also in the inactive parts of the body, leading to endothelium disorders [25–27]. To cause improvement of microcirculation reactivity and endothelium functioning, exercises of moderate intensity should last at least 30 minutes [28, 29]. This is why the aim of the study is to investigate whether 8-week aerobic training will improve skin microcirculation reactivity and endothelium functioning in patients with IHD, as well as whether potential improvement will last in the case of lack of physical activity.

## 2. Materials and Methods

Forty-eight men took part in the investigation with a stabilized and pharmacologically controlled ischaemic heart disease lasting at least a year, without acute coronary incidents in a medical interview. The coronary artery disease was diagnosed and confirmed by noninvasive methods—ergo testing. According to the CCS (Canadian Cardiovascular Society) classification, all of the subjects belonged to class 1, in view of the disease stage. None of the subjects smoked and they did not practise systematically any physical activity. Throughout the whole time of the experiment, the subjects took standard medicines and followed their foregoing life style. The subjects were asked not to drink any alcohol or not to use any other stimulants during the testing period. Women were excluded from the study because differences in the hormone economy induce differences in regular flows and the provoked ones [30].

The participants were randomly divided into two groups with 24 people in each of them. The research group participated in a aerobic march training. The march took place 3 times a week for approximately 30–40 minutes, over a period of 8 weeks. The subjects were marching with the intensity not exceeding 50% HR max, controlled on the basis “walk and talk.” After the time of training, the subjects did not practise any physical activity for the period of 8 weeks. Characteristics of the subjects are presented in Table 1.

According to the requirements of Helsinki Declaration, every subject was informed in detail about the aim of the study, the applied methodology, potential adverse effects, and a possibility of resigning from participation in the study at any stage of it, without giving reasons for this decision. The participants of the study expressed a conscious, written consent form for participation in the research. The experiment was accepted by the Bioethics Commission at the District Medical Chamber in Opole (No. 132).

### 2.1. Skin Microcirculation Measurement

Skin microcirculation measurement was conducted by using the Doppler laser flowmeter Perifluks 4001, produced by the firm Perimed (Sweden). This technique is noninvasive and allows flow registration in the real time, and it was described earlier [31].

Registration of flows in skin microcirculation was carried out in a continuous way, and it comprised the area of 1 mm^3^ under the probe. Data reading and the analysis of indicators were conducted by means of specialist software Perisoft for Windows.

All the investigations of skin microcirculation were performed in the same room by a trained person. The research was conducted in a lying position on the back, at the constant room temperature 21°C ± 1.2°C, with air humidity 40–60%, in the morning hours (between 8 and 12). The participants did not consume any drinks 8 hours before the investigation, which might have influenced microcirculation (tea, coffee, alcohol, coca-cola, and energy drinks) and, for at least 12 hours earlier, had not participated in an intensive physical activity [32]. During the experiment, the subjects were lying still, and they did not perform any sudden movements, as well as maintaining steady natural breathing rhythm [33].

The optode for measuring flows in skin microcirculation was placed on the skin of the back of the dominating hand, between I and II medial bone, by means of a disc adhesive on both sides. Before placing the disc, the skin was cleaned and disinfected with the use of colourless solution Softasept N and then left to dry [34]. The position of probe was described precisely in order to use the same position of the optode with each measurement. To avoid artefacts caused by movement of lower limbs, the forearm was stabilized carefully as well as fiberoptic cables were protected and fixed. Optodes were calibrated before each measurement, according to the recommendations of the producer. In the investigation, physiological stimulation was applied in the form of occlusive and thermal stimuli. The measurements were conducted three times: before starting the experiment: the initial measurement; after 8 weeks of aerobic training: measurement I; after 8 weeks of lack of physical activity: measurement II.

The course of the study is as follows:

1. The procedure was started in a patient after about 20 minutes of flow stabilization in the lying position.
2. Blood pressure measurement RR (mm·Hg) was conducted on the brachial artery in the dominating limb.
3. The regular flow (RF) in the lying position was registered in the dominating upper limb, and the time of the investigation was 4 minutes.
4. Flow in response to tightening on the arm of the pressure gauge cuff filled with air and to pressure higher than 50 mm Hg from the systolic pressure, measured earlier on the brachial artery was registered which is the so-called biological zero (BZ) and the time of the investigation was 4 minutes [31].
5. Hyperemic reaction in response to cuff loosening (PRH) was registered.
6. Flow was stabilized to the level of regular flow.
7. Optode temperature, using a heating module built in the probe, was increased up to 44°C. Heating time was 30 minutes. Temperature of the local heating unit integrated with the device was being increased at the pace 1°C × 10 s^−1^, to the temperature 44°C, and then for approximately 30 minutes remained at the same level, until it reached a stable phase of the blood stream (the so-called plateau). In none of the subjects did the increase of local temperature cause any pain. The feeling of pain causes release of neuropeptides, which independently of NO may influence the flow values.
8. Hyperemic reaction in response to temperature (TH) was registered.

Flow values were measured in relative units, on a conventional scale of perfusion units (PU, perfusion unit), proportional to the energy of the Doppler signal. Output voltage from the microprocessor corresponds to the blood flow in the unit of tissue volume (1 PU = 10 mV at the output) [31].

Vibration analysis within the blood flow is used to assess the control mechanisms of skin microcirculation *in vivo*. Assessment of periodical changes in the flow signal from the microcirculation area is enabled by the spectral analysis with the use of wavelet analysis or quick Fourier transformation; however, a clear surface of spectral components for low frequencies is achieved only in wavelet transformation, and this method was used in the present study [35–38].

Thanks to it, transformation of the temporal domain signal of the flow is achieved in the frequency spectrum in which the periodic activity amplitude is visible for each constituent frequency of the laser signal. For each frequency period, various lengths of the window were used, which prevented the phenomenon of the so-called “leakage” (frequency components in the spectrum escape to other frequencies). Frequency of signals which were achieved by means of laser Doppler flowmetry was analysed in the range from 0.01 to 2 Hz during the regular flow. In this range, five groups were distinguished: I: frequency band in the range of 0.01–0.02 Hz; II: frequency band in terms of 0.021–0.05 Hz; III: frequency band in the range of 0.051–0.145 Hz; IV: frequency band in the range of 0.15–0.5 Hz; V: frequency band in the range of 0.51–2 Hz. In each of the ranges, a different factor decides about the oscillation of blood. I presents vascular oscillations dependent on metabolic activity of endothelium (RŚ). Kvandal et al. [37] proved that, at least partly, this oscillation depends on NO. II presents the influence of the sympathetic nervous system on skin flow (RN) [39]. III illustrates oscillations resulting from the basic systolic tension of the arteriole, appearing as a result of particular myocyte discharge creating a circular layer of the vascular muscle; this reaction is often called myogenic and it is independent of the sympathetic nervous system. IV illustrates the respiratory rate. V illustrates the cardiac frequency. A time constant of 0.03 s was chosen, and the data were collected with the sampling frequency of 32 Hz [37, 38]. The signal strength and average amplitude in every range specify the spectral components [34].

Reading the value of individual indicators and the spectral analysis was conducted by means of Perisoft software. The files containing peaks too large in relation to the average (the so-called artefacts resulting from unpredicted movements) were eliminated, according to the recommendations of the producer and the Perisoft software capabilities [40]. Quantitative measurement of the blood flow is not possible to be applied in the Doppler technique due to significant individual differences in the structure and control of skin microcirculation. Therefore, we did not analyse the absolute values and we observed only the changes in relation to the resting values [41].

### 2.2. Statistical Analysis

The material was developed by the methods of descriptive statistics. The basic numerical characteristics of the variables under study were designated, the arithmetic average (*x*) and standard deviation (SD). Normal distribution of variables was verified by the Shapiro–Wilk test. In the case of lack of normal distribution, the data were logarithmed. Differences between the investigated indicators were assessed by means of the variance analysis with repeated measurements (ANOVA). To define differences between averages from individual groups, the post hoc test (Tukey test) of multiple comparisons was used. For the statistically significant difference, the level *p* < 0.05 was assumed.

## 3. Results

The values of microcirculation indicators obtained in the control group did not differ significantly between the measurements, which is why they were not shown in the present paper.

Skin microcirculation indicators in the research group are compared in Table 2.

In the first measurement in relation to the output values, a decrease of body mass was noted by about 2.21 kg on average and lowering of the systolic pressure and diastolic pressure by 10.4 mmHg and 3.68 mmHg respectively. The regular flow (RF) increased after the training by 20.21%. Two provoked reactions were as follows: hyperemic (PRHmax)–an increase occurred by 8.76%; hyperthermic (THmax)–an increase occurred by 5.38%. The time to achieve PRHmax by 42% and to achieve THmax by 22% were also reduced. Heart rhythm and the signal strength of the neurogenic rhythm also lowered by approximately 8% and 24%, respectively. The signal strength of endothelic rhythm increased by 19%. In measurement II, a recourse was noted in the values of researched indicators, which assumed values close to the output ones.

## 4. Discussion

In the study, a reactivity increase was demonstrated in skin microcirculation after 8 weeks of aerobic training of moderate intensity, in patients suffering from the ischaemic heart disease. After 8 weeks of lack of physical activity, a recourse occurred to the values noted before the training. Statistically significant changes were not revealed in the control group not participating in the physical activity, which may indicate that just aerobic training or its lack could decide in this study about changes in the skin microcirculation.

Because there is lack of standardised reference values for the skin vascular bed, both in relation to resting flows as well as for the values of provoked reactions, it is difficult to state which values of the indicators are correct (Freccero et al., 2006). It is assumed, however, that in patients with CAD, both the resting flows are lowered (RF) as well as provoked hyperemic reactions [10, 42–44]. It is also confirmed by the results achieved in the present study, in which the output values of the regular flows in the research group and the control group are lower than the ones obtained in other studies on healthy people and people active physically [45].

In the own study, it was revealed that after 8 weeks of training, a significant increase of RF occurred in the research group. According to Tinken et al. [18] and Hambrecht et al. [17], physical effort causes generalized increase of blood perfusion, which generates increase of shear stress and causes increase of production and/or release of vasodilatation factors (NO, EDHF) and/or greater sensitivity of smooth muscles to their action [30]. Some of the authors suggest that the hyperpolarizing factor is released only in the case of NO deficiency, because nitric oxide causes feedback inhibition on EDHF [46]. Malinowski et al. [47] stated that, however, in the case of microcirculation in small vessels, the hyperpolarizing factor, and not nitric oxide, regulates to a greater degree the vessel diameter. In the present study, however, it was not investigated which of the produced substances, NO or EDHF, is responsible for the increase of the values of resting flow. Hodges et al. [48] suggest that adaptation of skin microcirculation vessels is the result of activity of thermoregulatory mechanisms and not shear stress. However, Green et al. [22] proved that manipulating with tension and temperature, heat per se does not cause any changes and to increase the vessel diameter, shear stress is needed generated by aerobic training.

It is possible that the increase in resting flows in the research group occurred as a result of body mass reduction by 2.2 kg on average. van der Heijden et al. [49] proved that higher BMI correlates with endothelium dysfunctioning in patients with suspected IHD. Colberg et al. [50] revealed dependence between the increase of basic perfusion and body mass loss. Hamdy et al. [51] stated that disorder of endothelium functioning may be caused by obesity and body mass loss causes improvement of functioning of vascular lining. The increase of resting flows may also have been influenced by regulating arterial blood pressure. The values of RR measured before the training were clearly raised in both groups, which correlate positively with dysfunctioning of microvessels in the skin [8]. After 8 weeks of activity, both the systolic pressure as well as diastolic pressure lowered significantly, which may have been the cause of RF improvement.

Postocclusive hyperemic reaction (PRH) is a transitional increase of blood flow after forced ischaemia. It is used as an instrument to investigate microcirculation functioning of peripheral vascular diseases [52]. Lowering PRH is identified with dysfunction of the vascular endothelium [53]; however, it is disputable, deficiency of which factor produced by endothelium is decisive. Cracowski et al. [54] believes that smaller production of EDHF is the cause; however, recently it was suggested that it is the lack of prostacyclin which causes lowering of PRH [11].

In the present experiment, lowering of maximum values of PRH (PRHmax) was noted as well as prolonging the time needed to reach the highest values of hyperemic reaction (*t*) in patients with IHD, in relation to values presented by other authors in healthy people [10, 42–44, 55–58]. Similarly, Ç Sim [11] observed lowered values of hyperemic reaction to occlusion in patients with ischaemia of the heart. In the latest reports, correlation is indicated of lowered PRH values with the risk of acute cardiovascular incidents [4, 55]. After 8 weeks of training in the research group, there appeared a statistically significant increase of maximum PRH values interpreted as an improvement of endothelium functioning.

It was stated that time parameters are a strong indicator differentiating groups of the ill and healthy people. Prolonging the time for reaching PRHmax (*t*) in patients with CAD was noted by Agarwal et al. [59]; and in the research of Shamim-Uzzanam et al. [55], *t* was almost twice as long as in healthy people. Similar results were revealed in the present study; however, after the aerobic training in the research group, significant reduction of time was demonstrated for achieving maxPRH. Time to reach maximum hyperemia is dependent on the body mass [60], and perhaps, this reduction of body mass in the research group had an influence on the reaction time. The pace of changes of the vessel diameter is also dependent on the reaction between peroxide anions and the factors produced by endothelium which expand the vessels [61]. Thus, increase in the antioxidant potential, as a result of aerobic training, may also decide about shortening the time *t*.

Hyperemic reaction in response to temperature (TH) runs in two stages—the so-called axon reflex is responsible for the flow increase in the first 2-3 minutes after turning on the heating element. However, the increase of value in the second phase (plateau), 20–30 minutes later, depends on NO and EDHF in the proportions, respectively, 70% and 30% [62]. The output values of the maximum hyperthermic reaction (THmax), in the present study, in both groups, the research and the control one, were lower than in healthy people. Similar results were obtained in [11]. After 8 weeks of aerobic training, significant increase of maximum values of the thermal reaction was noted in phase II, which may suggest improvement of vasodilators release (NO, EDHF) and/or more efficient response of smooth muscles to these compounds. Tew et al. [63] observed that regular aerobic exercises may increase the peak values of thermal reaction, which they explain by the increase in the production of mediators and an intensified response of the vessels to these transmitters. Taddei et al. [64] did not reveal any increase in the values of the thermal reaction as a result of training. Methodological differences may have influenced such results but also the duration time and intensity of the exercises. Goto et al. [65] proved that only training of moderate intensity (50% VO_2_max) causes improvement of vasodilation. High intensity (75% VO_2_max) causes an increase of postexertional oxidative stress or intensification of arginase activity, which may determine lowering of the values in the plateau phase [66]. Reaching the peak of hyperthermic reaction (tt) in healthy people lasts approximately 3 minutes [30]. In the present experiment, it was demonstrated, that in all the subjects, the time needed to reach the peak of hyperthermic reaction was longer than 3 minutes, and in the research group, after the period of physical activity, it decreased significantly.

Changes in the oscillation amplitudes provide information about the dynamics of control mechanisms in the skin microcirculation. The higher the amplitude of oscillation, the stronger the influence on perfusion [36]. A significant increase of the signal strength in the range of endothelial oscillation and a significant reduction of signal strength in the neurogenic frequency as well as heart rhythm were noted in the group which was exercising. Lowering the heart rate (HR) frequency after endurance training is a recognised fact, and heart rhythm (RS) constitutes here a reflection of heart rate in the skin microvessels [67]. An increase in the values of signal strength in the frequency range 0.01 Hz in the research group after training indicate that endothelium releases greater amounts of NO or the vascular smooth muscles react stronger to this factor. A weaker neurogenic stimulation occurs, which may also be the cause of increase in posttraining values.

The results of the study demonstrate a clear improvement of endothelial functioning measured in LDF, after aerobic training. However, after 8 weeks, in which the subjects returned to the lifestyle without physical activity, the indicators of skin microcirculation assumed the values close to the output ones, which were registered before starting the training. Probably, improvement of endothelial functioning is temporary and only takes place with systematic exercises. This is also confirmed by the results of Bleeker et al. [68]. The improvement effects of endothelial functioning may be achieved pharmacologically (antihypertensive treatment, statins, and metformin); however, it is interesting that the positive changes take place only during treatment, and after stopping of the treatment, they recede, similarly as in the case of improvement occurring after the training [69].

Improvement of endothelium functioning seems to be crucial in reducing the cardiovascular risk, and it is considered to be the basic cardioprotective mechanism [7, 62]. In connection with the above, it can be considered whether the aerobic training of moderate intensity should be a standard in prevention and treatment of IHD. It should be, however, pointed out that such activities should not be periodical but they ought to be systematic and continuous.

Diseases coexisting in patients with IHD were a limitation of the present study (diabetes, hypertension, metabolic syndrome, and dyslipidemia) which may have influenced the research results. However, it is difficult to select a group with ischaemic disease, and without any other diseases, which most frequently constitute IHD risks and are closely related to each other. Only in 6 subjects no other diseases were noted, and the results would not be reliable.

Involving in the study only male patients was another limitation of the present study. Recruiting the research and control group from one population was intended, because the influence of the menstruation cycle phases was revealed, and, therefore, the influence of female hormones on the vascular core might have disturbed the results [30, 70]. However, the theme still remains not exhausted and it indicates the directions of further research.

## 5. Conclusions

1. 8 weeks of aerobic training causes significant improvement of endothelium functioning, expressed in reactivity improvement in skin microcirculation in patients suffering from ischaemic heart disease. It indicates aerobic training as an effective nonpharmacological, cardioprotective factor.
2. The improvement effects of skin vascular bed functioning in the group of patients with IHD are unstable and disappear after the time in which the patients did not practise physical activity.

## Figures and Tables

**Table 1:** Characteristics of the subject.

Index					
*X* ± SD	Age (years)	Height (cm)	Body mass (kg)	BMI (kg/m^2^)	Percentage body fat (%)
The research group	63.04 ± 4.65	178.53 ± 6.38	104.51 ± 8.97	32.8 ± 6.26	27.16 ± 11.76
The control group	65.02 ± 5.16	174.33 ± 3.35	105.84 ± 12.83	34.6 ± 5.79	29.43 ± 6.2

*X* ± SD: mean (*x*) ± standard deviation (SD).

**Table 2:** Skin microcirculation indicators in the research group.

Indicators	Output values	I measurement	II measurement
Body mass (BM) (kg)	104.51 ± 14.45	102.3 ± 14.36^*∗*^	104.69 ± 14^2^
Fat content (*F*) (%)	32.92 ± 13.95	27.16 ± 11.76^*∗*^	29.84 ± 1.62
Systolic pressure (SBP) (mm·hg)	154.22 ± 14.92	143.82 ± 14.94^*∗*^	147.47 ± 15.72^2^
Diastolic pressure (DBP) (mm·hg)	105.01 ± 13.65	101.33 ± 13.23^*∗*^	103.36 ± 2.98^2^
Resting flow (RF) (PU)	11.43 ± 1.53	13.75 ± 2.54^*∗*^	12.25 ± 2.22^2^
Biological zero (BZ) (PU)	2.86 ± 0.34	2.85 ± 0.29	2.8 ± 0.24
Postocclusive hyperemic reaction, maximum values (PRH_max_) (PU)	53.05 ± 10.6	57.70 ± 6.55^*∗*^	55.65 ± 10.63^2^
Time to reach PRH_max_ (*T*) (s)	19 ± 0.18	11 ± 0.13^*∗*^	18 ± 0.11^2^
Hyperemic reaction to temperature (TH), maximum values (TH_max_) (PU)	80.01 ± 14.53	84.96 ± 11.5^*∗*^	82.59 ± 18.35^2^
Time to reach TH_max_ (TT) (s)	263 ± 12.64	205 ± 11.72^*∗*^	222 ± 10.57
Heart rhythm (cycle × minute^−1^)	78.89 ± 3.87	72.9 ± 2.94^*∗*^	76.95 ± 1.66^2^
Heart rhythm signal strength (PU^2^ × Hz^−1^)	0.35 ± 0.22	0.32 ± 0.19	0.35 ± 0.3
Breathing rhythm (cycle × minute^−1^)	13.92 ± 1.15	13.82 ± 1.04	13.95 ± 0.61
Breathing rhythm signal strength (PU^2^ × Hz^−1^)	0.59 ± 0.14	0.58 ± 0.17	0.53 ± 0.17
Myogenic rhythm (cycle × minute^−1^)	5.89 ± 0.37	5.85 ± 0.34	5.88 ± 0.42
Myogenic rhythm, signal strength (PU^2^ × Hz^−1^)	1.15 ± 0.81	1.09 ± 0.76	1.13 ± 0.63
Neurogenic rhythm (cycle × minute^−1^)	2.09 ± 0.37	2.05 ± 0.34	2.1 ± 0.36
Neurogenic rhythm, signal strength (PU^2^ × Hz^−1^)	2.89 ± 1.15	2.18 ± 0.87^*∗*^	2.7 ± 0.45^2^
Endothelial rhythm (cycle × minute^−1^)	0.9	0.9	0.9
Endothelial rhythm, signal strength (PU^2^ × Hz^−1^)	0.91 ± 0.2	1.09 ± 0.27^*∗*^	0.97 ± 1.19^2^

^*∗*^Statistically significant difference in relation to measurement I. ^2^Statistically significant difference in relation to measurement II.

## Data Availability

The datasets generated during and/or analysed during the current study are available from the corresponding author on reasonable request.
